# Understanding the support for gender-based harassment perpetrators: the role of closeness and empathy

**DOI:** 10.3389/fpsyg.2024.1418404

**Published:** 2024-06-27

**Authors:** Nira Borges-Castells, Verónica Betancor, Armando Rodríguez-Pérez

**Affiliations:** Department of Cognitive, Social and Organizational Psychology, Faculty of Psychology, University of La Laguna, Santa Cruz de Tenerife, Spain

**Keywords:** closeness, empathy, gender-based harassment, moral justification, dehumanization

## Abstract

**Introduction:**

Gender-based harassment is a pressing social challenge urgently demanding eradication. While social movements emphasize supporting victims, societal responses sometimes lean toward exculpating perpetrators. This study examines two factors influencing this exoneration: closeness to perpetrators and empathy focus.

**Methods:**

A total of 345 participants took part in an experimental design to assess how closeness to perpetrators (close vs. distant) and empathy focus (on the perpetrator vs. victim vs. control) impact the moral justification of harassment and the dehumanization of both parties.

**Results and discussion:**

Results indicate that closeness and empathizing with perpetrators lead to greater leniency—more moral justification and less dehumanization of the perpetrator. Heightened moral justification for close perpetrators is mediated by increased empathy toward them and decreased empathy for victims, and reduced dehumanization of close perpetrators corresponds to heightened empathy toward them. This research highlights how closeness and empathy, two initially positive factors, can foster tolerance toward gender harassment.

## 1 Introduction

Despite widespread global efforts to combat gender-based violence, it remains a severe issue that poses challenges to social justice, public health, and the legal system (Standish, 2014). In response to the urgency of addressing this challenge, movements such as “Sister, I do believe you (Hermana, yo sí te creo)” have emerged (Angulo Egea, 2019). This movement symbolizes the importance of supporting and standing by victims, acknowledging their courage in sharing their experiences and challenging the prevailing culture of victim blaming. Despite such support for movements that emphasize the importance of backing victims, however, victims are more commonly blamed than perpetrators.

Various psychological and social factors that have received considerable scholarly attention contribute to this phenomenon. System justification theory (Jost and Banaji, 1994) posits that people have a psychological need to defend and justify existing social, economic, and political systems. This need fosters attitudes and behaviors that maintain the status quo, including victim-blaming. Beliefs in a just world (Lerner, 1965) further exacerbate this phenomenon, as individuals who hold these beliefs tend to think that people get what they deserve. As a result, victims of harassment are often perceived as deserving their fate, while perpetrators are exonerated. Research supports this, showing that individuals with strong just world beliefs are more likely to blame victims and excuse perpetrators of gender-based violence (Valor-Segura et al., 2011). Additionally, sexism and gender stereotypes reinforce these tendencies. Sexism is associated with a tendency to blame the victim under certain conditions (Durán et al., 2010), and hostile sexism is a key predictor of these attitudes, with individuals holding such beliefs particularly inclined to justify discrimination and aggression against women (Valor-Segura et al., 2011). Moreover, gender system justification can also lead to the perception of blame on both the victim and the perpetrator (Murray et al., 2023).

In this study, we will focus on other factors that have not been as thoroughly explored. Specifically on how closeness and the focus of empathy—two virtues initially intended to facilitate care and understanding—can become factors that facilitate the defense of perpetrators, thus blurring moral lines. Specifically, we assess how closeness to the perpetrator and the focus of empathy toward the perpetrator impact moral justification and the dehumanization of both the perpetrator and the victim.

All living beings share the feeling of profound affection for those around us, fueling an unwavering commitment to protect and defend them. The significance of relationships in one’s close circle is such that it can provoke differences in emotional and behavioral responses. For example, the human brain exhibits more reactions to the pain experienced by a loved one compared to that of a stranger (Cheng et al., 2010). In the same vein, we also lean toward partiality when it comes to experiencing empathy. From childhood, we are biased toward feeling greater empathy for individuals in our close circle (e.g., mother) than toward strangers (Davidov et al., 2013). This inclination to feel more concern and empathy toward individuals in our immediate environment may have a positive aspect, since it aligns with our human need for social cohesion and survival. However, there are situations where this inclination might not be advantageous. Our loved ones, being human, are susceptible to engaging in negative and immoral behavior. In such instances, a dilemma arises: do we persist on the path of unconditional loyalty and love, or do we take a stand and express disapproval?

The scientific literature indicates that people tend to lean more toward the first option: they are more forgiving of the people they are close to when they engage in immoral or negative actions. Closeness to the transgressor of negative actions can elicit a more benevolent evaluation of the transgression (Gino and Galinsky, 2012; Forbes and Stellar, 2022), a greater moral rationalization (Forbes and Stellar, 2022), and a tendency to protect rather than report the transgressor (Weidman et al., 2020; Berg et al., 2021). More relevantly for this research, such defense of perpetrators who belong to their close circle also occurs in cases of gender-based harassment (Borges-Castells et al., 2024). Specifically, people perceive harassment as less negative, justify it more, and dehumanize the perpetrator less when the perpetrator is someone close (e.g., a friend or family member) rather than someone unknown or distant. This circumstance is of significant concern, as it may contribute to the perpetuation of gender-based violence. According to Waltermaurer (2012), the community’s perception of gender-based violence strongly shapes responses toward violence, affecting whether the violence occurs, if victims report it, and if third parties speak out. In a society that defends perpetrators through justifications, the likelihood of continued aggression rises. This could especially be the case when there is closeness to the perpetrators. In this article, we reexamine whether individuals are more lenient toward close perpetrators: we aim to determine whether the perpetrator is more justified and less dehumanized, and whether the victim is more dehumanized, when the perpetrator is someone close. Moreover, our goal is also to examine the role of empathy in the response to gender harassment.

Empathy is a complex, multidimensional construct with distinct components—affective, motivational, and cognitive—that operate in parallel (Decety and Jackson, 2004; Decety and Svetlova, 2012). Scientific literature emphasizes the importance of distinguishing each of the empathy facets to avoid vague conceptualizations from the broad use of the term (Decety and Cowell, 2014). In this article, our focus is on the motivational aspect of empathy, known as empathic concern, which entails caring for another person’s well-being. Consequently, when we use the term “empathy,” it specifically denotes empathic concern. Recognizing the significance of empathy is crucial when addressing gender-based violence because empathy can foster moral and positive behavior. For example, it provides commitment to end a victim’s suffering, transcending considerations of group membership and social hierarchies (Decety and Cowell, 2014); it plays a vital role in effectively preventing sexual and gender-based violence in its primary stages (Gevers and Dartnall, 2014); and programs based on fostering empathy toward the victim are effective among men at high risk of committing sexual abuse (Schewe and O’Donohue, 1993).

Although the concept of empathy normally implies promoting positive and moral actions, it can also serve to justify and endorse immoral behavior. Particularly when directed toward one’s offspring or members of one’s social group, empathy possesses certain unfortunate features that may directly clash with moral behavior (Decety and Cowell, 2014). Furthermore, in the context of behaviors associated with gender-based harassment, existing literature highlights the importance of considering the empathy felt for both the victim and the perpetrator. Research has indicated that individuals are more likely to endorse myths about sexual harassment when considering the scenario from the perspective of the perpetrator rather than that of the victim (Diehl et al., 2014). Furthermore, Bongiorno et al. (2020) examine the influence of feeling empathy toward the perpetrator or the victim on men and women’s responses when evaluating sexual harassment behavior, finding that men tend to blame women more for being sexually harassed, and this blame is mediated by a greater sense of empathy toward the perpetrator. Therefore, in this article we consider that empathy could be a double-edged sword: on the one hand, it could help prevent gender-based violence; on the other hand, it could help perpetuate it.

In light of these precedents, we propose that closeness to and empathizing with the perpetrator may lead to increased leniency toward harassment. By leniency, we specifically refer to three reasoning processes involved: more moral justification, less perpetrator dehumanization, and more victim dehumanization. These variations in moral justification and dehumanization, associated with closeness and empathy, could raise significant concerns.

First, moral justification involves finding reasons or arguments that support the morality of an action that is considered immoral and negative (Bandura, 1991; Tsang, 2002). When people engage in moral justification, they are able to violate their moral standards because they have convinced themselves that this behavior is not immoral. When moral justification is applied to a behavior of gender-based violence, it poses significant challenges. Research indicates that the justification of gender-based violence contributes to the persistence of such behaviors (Waltermaurer, 2012). Furthermore, it may help legitimize gender inequality, reinforcing support for harmful beliefs in the fight against gender violence. For example, it can provoke an increased acceptance of myths about sexual assault (Chapleau and Oswald, 2014).

Second, closeness and empathy could lead to lesser dehumanization of the perpetrator. Dehumanization is the process by which individuals attribute fewer mental and perceptual capabilities to a person, considering them less human (Gray et al., 2007). In this study, we specifically focus on de-mentalization, which is one aspect of the process through which individuals dehumanize others. It entails denying individuals the capacity to feel, experience, or, in terms of agency, engage in rational activities (Gray et al., 2007). Existing literature demonstrates that perpetrators can also be subject to dehumanization. Specifically, individuals dehumanize those who have committed crimes (Bastian et al., 2013) and those who have caused harm to others (Khamitov et al., 2016). For instance, some perpetrators may be labeled as monstrous (Farr, 2000; Vasquez et al., 2014) or are believed to possess animalistic cravings for crime (Jahoda, 1999; Haslam, 2006). In our research, we propose that closeness and empathy may attenuate this dehumanization process toward perpetrators. We argue that when perpetrators are close and empathy is focused on them, they may not be dehumanized but, rather, humanized by attributing more agency to them. This, in turn, could imply greater leniency toward their actions.

Third, victims could be more dehumanized. It has been demonstrated that viewing victims as less human can lead to several negative outcomes. For example, it can lead individuals to provide them with less assistance (Cuddy et al., 2007), it can serve as a rationale for displaying increased aggression toward them (Bandura et al., 1975), and it can lead to significant cognitive and emotional outcomes, including unfavorable self-awareness, feelings of shame and guilt, and disorganized cognitive states and emotions of sadness and anger (Bastian and Haslam, 2011). In this sense, it is crucial to consider the potential factors that may result in the dehumanization of victims—such as closeness to the perpetrator and empathy toward him—to avoid such adverse consequences.

### 1.1 The present research

We investigate how closeness to perpetrators and empathy focus impact the moral justification of harassment behavior, as well as the mind attribution of both the perpetrator and the victim. Moreover, we delve into the relationship between closeness and empathy. Therefore, our first two hypotheses are the following:

- H1: When the participant has a close relationship (vs. distant) with the perpetrator, regardless of the empathy focus (perpetrator vs. victim vs. control condition), the participant will justify the perpetrator’s behavior more (H1a), dehumanize the perpetrator less (H1b), and dehumanize the victim more (H1c).
- H2: When the participant has a distant relationship (vs. close) with the perpetrator, there will be differences depending on the empathy focus. Specifically, when empathy is focused on the perpetrator, the participant will justify the perpetrator’s behavior more (H2a), dehumanize the perpetrator less (H2b), and dehumanize the victim more (H2c).

Moreover, our aim was to consider empathy not only as a causal factor but also as a potential mediator of responses to harassment. Previous research indicates that individuals tend to morally justify harassment behavior more when it is carried out by someone known rather than by a stranger (Borges-Castells et al., 2024). However, the underlying mechanism in this process remains unknown. Consequently, we propose the following hypothesis:

- H3: The propensity to use justification strategies when the perpetrator is close could be explained by feeling less empathy toward the victim (H3a) and feeling greater empathy toward the perpetrator (H3b).

All hypotheses have been pre-registered and can be found at this link: https://osf.io/zek7f. Additionally, the databases and materials used in the research are available at this link: https://osf.io/whta4.

## 2 Method

### 2.1 Participants

Our sample consisted of 345 participants, all residents in the United Kingdom. They were recruited through Prolific and paid $1.10 for participating. Gender was largely balanced between women (47.2%) and men (52.8%). The average age was 30.89 (*SD* = 5.53), ranging from 18 to 45 years old. In terms of education, most participants had a university-level education (41.7%) or a postgraduate education (24.9%). To determine the required sample size, we conducted an *a priori* power analysis using the software program G*Power 3.1 (Faul et al., 2007). Our goal was to obtain 0.95 power to detect a medium effect size of 0.25 at the standard 0.05 alpha error probability. G*Power recommended a sample size of 323 participants to meet this goal.

### 2.2 Data exclusion

Our initial sample comprised 439 participants. However, we excluded 94 participants from the analysis based on specific criteria. First, we omitted participants that failed any of the attention checks. Second, we removed participants who could not imagine a man with the characteristics we requested to conduct the survey. Third, to ensure that the conditions accurately reflected our manipulation, we followed the insights from Gächter et al. (2015), who noted that a score of 2 or less on the Inclusion of the Other in the Self scale (IOS; Aron et al., 1992) traditionally signifies acquaintances. Therefore, in conditions where participants were intended to be close with the perpetrator, we excluded those with a score of 2 or lower on this scale. In conditions where participants were intended to be perceived as distant with the perpetrator, we excluded those with a score of 3 or higher. This was necessary to check the perceived closeness of the selected individuals.

### 2.3 Design and procedure

We followed a between-subjects experimental design with 2 (closeness to the perpetrator: close vs. distant) and 3 levels (empathy focus: perpetrator vs. victim vs. control condition). The surveys were constructed on the Qualtrics platform. Participants were initially presented with a consent form outlining the voluntary and anonymous nature of their participation in the experiment and assuring them that no harm would come from their engagement in the study. The consent form also specified the source of funding for the experiment. Afterwards, participants were asked to provide sociodemographic information, including their age, gender, and educational background. Following the demographic data collection, participants were directed to the main experiment. They were randomly assigned to one of six conditions, each differing in two key aspects: the closeness of the participant and the perpetrator (close vs. distant) and where the empathy focus was directed (perpetrator vs. the victim vs. control condition).

In the close perpetrator condition, participants were told to think about a man they loved and knew well, someone who liked women and was around their age. In the distant perpetrator condition, the instructions were the same, except they were asked to consider a man they only knew by sight. Subsequently, they were asked to confirm their ability to imagine a man with those characteristics. If they could not, they were excluded from the questionnaire. Those who were able to do so then responded to a question aimed at assessing their closeness to the chosen individual. We used the Qualtrics “piped text” function so that the name they indicated appeared in the rest of the questionnaire.

Afterward, they were shown a description of a gender harassment behavior performed by a man (close or distant) toward a woman. Following this, a forensic psychological report was introduced, which depicted the harassment behavior from either the perspective of the victim or the perpetrator, while also outlining the psychological consequences the situation had for one of them. The purpose of this report was to evoke empathy for either the perpetrator or the victim. In the control condition, participants were not presented with any report; only the harassment behavior was shown. Then, participants responded to a scale of empathy toward the man and toward the woman and assessed the wrongness of the behavior.

Subsequently, they answered questions related to the dependent variables, including moral justification and dehumanization of the man and the woman. After these assessments, participants responded to a scale of modern sexism. Finally, they were fully debriefed, receiving clarification that the scenario presented in the research was fictitious, was not based on real events, and did not involve real individuals.

### 2.4 Materials

#### 2.4.1 Closeness assessment

We used the IOS scale from Aron et al. (1992). This pictorial tool asks participants to assess their connection with a particular individual by choosing one out of seven pairs of circles that gradually overlap (from 1 to 7). Participants were asked to select the pair of circles that most accurately represented their relationship with the man they have chosen, understanding that greater separation between the circles indicated a weaker connection, while closer proximity meant a stronger connection.

#### 2.4.2 Transgression

Gender-based violence, as defined by the United Nations General Assembly (1993), includes any act resulting in, or likely to result in, physical, sexual, or psychological harm to women. This article focuses on gender-based harassment which is the most prevalent form of sexual harassment (Leskinen et al., 2011; National Academies of Sciences, Engineering, and Medicine et al., 2018), particularly addressing the common occurrence of men perpetrating harassment against women (Fitzgerald and Cortina, 2018). More specifically we address stalking, which is considered a manifestation of technology-facilitated intimate partner violence (TFIPV; Attrill-Smith and Wesson, 2020). We chose to examine a TFIPV behavior due its emergence as a novel form of gender-based violence with the advent of new technologies (Gámez-Guadix et al., 2015). Participants were presented with a scenario where a woman reported her boyfriend (the perpetrator) after discovering the harassing behavior he had exerted against her: “A woman has reported [name of the close vs. distant perpetrator] after discovering he had downloaded an application to track her cell phone without her consent.” We selected this behavior because it represents a common form of online gender violence against women—according to Women’s Aid (2014, 2019), in a minimum of 29% of cases involving domestic or intimate violence, the partner or ex-partner employed spyware or geolocation devices installed on the victim’s computer or mobile phone.

#### 2.4.3 Empathy focus manipulation

To elicit empathy toward either the perpetrator or the victim, we developed a forensic psychological report. This report illustrated either the woman’s perspective on her experience of harassment (“When I went out with my friends, he insisted on knowing where I was, and if I did not respond quickly, he would get angry with me”) or the man’s perspective (“She would always hang out with some new friends, and I started to think negatively. I would ask her where she was going, and she either would not answer or took a long time to respond”). Additionally, both reports depicted the psychological consequences of the experience for them—the woman, due to the harassment she suffered, and the man, due to facing accusations of harassment (the full reports are provided in the Supplementary material). We chose to adopt this procedure loosely based on the approach used by Diehl et al. (2014), who provided participants with personalized reports featuring either a female target of workplace sexual harassment or the alleged male perpetrator.

Given that immersing oneself in another person’s situation and focusing on their emotions often elicits feelings of empathic concern (Batson et al., 1997; Galinsky and Moskowitz, 2000; Batson et al., 2002), our manipulation aimed to prompt participants to adopt the perspective of the perpetrator or the victim and, consequently, generate empathic concern. We conducted a pilot study (*N* = 60) to confirm the effectiveness of the manipulation. A significant interaction was found between empathy manipulation and feeling empathy toward the man and the woman: *F*(58,2) = 14.35, *p* < 0.001, *η_p_*^2^ *=* 0.19. We found that participants exhibited greater empathy toward the woman when empathy was focused toward the victim (*M =* 6.04, *SD =* 0.83), as opposed to when it was activated for the perpetrator (*M =* 5.13, *SD =* 1.49, *p <* 0.001). Also, participants exhibited greater empathy toward the man when empathy was focused on the perpetrator (*M =* 3.21, *SD =* 1.21) rather than on the victim (*M =* 2.32, *SD =* 1.10, *p <* 0.001). The distribution of these results aligns completely with that of Bongiorno et al. (2020), who tested the manipulation effectiveness in the same way. Moreover, we confirmed that both stories were equally imaginable: *F*(59,1) = 0.50, *p =* 0.481, *η_p_*^2^ = 0.009.

#### 2.4.4 Empathy toward the man and the woman

We measured empathy to assess whether the manipulation of the empathy focus was effective and to use it as a part of our mediation analysis. This assessment involved measuring empathy toward both the perpetrator and the victim in all conditions using the empathic concern scale (Batson et al., 1997; Bongiorno et al., 2020). The scale comprises four items related to empathy: empathy, concern, sympathy, and compassion, along with distractor items. The scale ranges from 1 to 7, with 1 being “I have not felt this way at all” and 7 being “I have felt this way completely.” Both items of empathy toward the man (*α_man_* = 0.81) and the woman (*α_woman_* = 0.91) showed a great internal consistency.

#### 2.4.5 Behavior wrongness

These measures aim to confirm that the selected harassment behavior is perceived as inappropriate and serious by the participants. Participants were asked to rate the extent to which they believed the behavior performed by the individual was inappropriate (1 = *Not inappropriate at all*, 7 = *Completely inappropriate*) and the extent to which they believed the behavior was serious (1 = *Not serious at all,* 7 = *Totally serious*).

#### 2.4.6 Moral justification

To assess moral justification, we followed the methodology employed by researchers such as Pina et al. (2021). We developed a scale of 8 items that represent the forms of justification derived from Bandura’s (1991, 2011) moral disengagement theory. More precisely, we generated a set of 6 items representing potential rationales for justifying the actions of the harasser (i.e., “It’s understandable that he did that; perhaps he felt his relationship was in jeopardy”; *α =* 0.91). The scale ranges from 1 = *Not justified at all* to 7 = *Completely justified*.

#### 2.4.7 Dehumanization of the perpetrator and the victim

To assess dehumanization, we used the Mind Attribution Scale by Gray et al. (2007). Specifically, we employed the abbreviated version, as implemented by Bernard et al. (2020), which involves selecting items most representative of each subscale. For agency, we chose items with the highest loadings on the agency factor and the lowest loadings on the experience factor (self-control, morality, and memory) from the original study (Gray et al., 2007). The same criterion was applied to the experience subscale (hunger, fear, and pain).

#### 2.4.8 Modern sexism

Finally, we assessed modern sexism (Swim et al., 1995) to examine its potential impact on responses to the dependent variables. An example item from this scale is: “It is rare to see women treated in a sexist manner on television.” The scale also follows a 1 to 7 range, where 1 = *Totally disagree* and 7 = *Totally agree*. The scale showed a great internal consistency (*α* = 0.89).

## 3 Results

### 3.1 Analytic strategy

We used the SPSS program for the analysis. A significance level of 0.05 was set. First, as we indicated in the pre-registration, we carried out several 2 × 3 ANOVA design with both factors between subjects to test H1 and H2. Additionally, we carried out a mediation analysis to examine the relationships described in H3. We carried out bivariate correlations and ANOVAs as exploratory analyses to test how gender and sexism were related to both the justification of harassment and dehumanization. We also conducted a mediation that was not pre-registered to gain a more comprehensive view of the data. Our aim was not to test additional hypotheses but to acquire more concrete and enriching insights from the dataset, thereby expanding our overall understanding of the subject without compromising the pre-registered hypotheses. The specific rationale for adding this analysis is provided in the results.

### 3.2 Manipulation check

First, we needed to ensure that close and distant perpetrators were perceived with a different degree of closeness. A *t*-test was conducted to examine the perpetrator’s closeness across experimental conditions. It revealed a significant difference (*t*(343) = 32.55, *p <* 0.001, *d = −*3.53 95% CI [−3.872 – −3.197]) between the close perpetrator condition (*M =* 4.69, *SD =* 1.23) and the distant perpetrator condition (*M =* 1.40, *SD =* 0.51).

Second, we needed to verify the effectiveness of the empathy manipulation. To do so, we conducted two one-way ANOVA analyses with three levels (empathy focus: perpetrator vs. victim vs. control) for both empathy toward the man and toward the woman. For empathy toward the man, significant differences emerged (*F*(2,344) = 34.12*, p* < 0.001, *η*_p_^2^ = 0.16). When empathy was focused on the perpetrator, participants felt significantly more empathy for the man (*M =* 4.04, *SD =* 1.41) compared to when the focus was on the victim (*M =* 2.62, *SD =* 1.16; *p* < 0.001) and to the control condition (*M =* 3.40, *SD* = 1.32; *p* < 0.001). For empathy toward the woman, we also found significant differences (*F*(2,344) = 16.29, *p* < 0.001, *η*_p_^2^ = 0.08). Empathy for the woman was higher when the focus of empathy was on the victim (*M =* 5.62, *SD =* 1.09) and in the control condition (*M =* 5.51, *SD =* 1.43; *p* = 1.00) compared to when the focus was on the perpetrator (*M* = 4.68, *SD =* 1.51; *p* < 0.001). These results support the effectiveness of the manipulation, aligning not only with the outcomes observed in the pilot study but also with those reported by Bongiorno et al. (2020), who identified a similar data distribution in their research.

Third, we needed to check whether the participants perceived the behavior as being wrong. To do this, two one-sample *t*-tests were conducted. The results showed that the inappropriateness and seriousness of the behavior were significantly different from the midpoint of the scale (*M =* 5.60, *SD* = 2.09, *p <* 0.001 for inappropriateness; *M* = 5.91, *SD =* 1.25, *p <* 0.001 for seriousness). These results indicate that participants indeed considered the harassment behavior to be both inappropriate and serious.

### 3.3 Main analysis

#### 3.3.1 Moral justification

To verify the results for moral justification, we carried out an ANOVA 2 (closeness to the perpetrator: close vs. distant) × 3 (empathy focus: perpetrator vs. victim vs. control) design with both variables between subjects. We found a significant main effect of closeness to the perpetrator (*p <* 0.001). When the perpetrator was close, participants used more moral justification strategies than when he was distant. Also, there was a significant effect of empathy focus (*p <* 0.001). Specifically, there was greater moral justification when empathy was focused on the perpetrator than when empathy was focused on the victim (*p <* 0.001) or in the control condition (*p <* 0.001). There were no significant differences between focusing empathy on the victim and the control condition (*p* = 0.353), and there were no significant interactions between closeness to the perpetrator and the empathy focus (*p =* 0.635). The ANOVA statistics, as well as the means and standard deviations of the main effects, can be found in Table 1.

**Table 1:** ANOVA statistics and descriptive statistics of the main effects.

Measure	Closeness				Empathy				
	*Close*	*Distant*	*F*(1,344)	η_p_^2^	*Perpetrator*	*Victim*	*Control*	*F*(2,344)	η_p_^2^
Moral justification	2.53 (0.09)	2.02 (0.08)	16.91***	0.048	2.78 (0.11)	1.91 (0.10)	2.14 (0.10)	17.17***	0.092
Perpetrator dehumanization	4.71 (0.13)	3.39 (0.12)	50.72***	0.13	4.26 (0.16)	3.70 (0.15)	4.18 (0.16)	3.62*	0.021
Victim dehumanization	5.43 (0.09)	5.39 (0.09)	0.08	0.00	5.11 (0.12)	5.61 (0.11)	5.52 (0.11)	4.95**	0.028

Figures outside parentheses represent means, and figures within parentheses represent standard deviations. Asterisks adjacent to *F*-values denote statistical significance: *p = 0.028, **p = 0.008, ***p < 0.001.

These results support H1a, which expected that when the perpetrator was close, participants would justify their actions more than when he was distant, regardless of the empathy focus. However, they do not provide support for H2a, which expected that when the perpetrator had a distant relationship with the participant, and empathy was focused on the perpetrator, the behavior would be more justified. That is because, even though we found a significant main effect of empathy focus, we did not find significant interactions. This means that the effect of empathy focus does not vary based on closeness to the perpetrator. Participants justify the perpetrator more when empathy is focused on him, regardless of the closeness that exists between them.

Furthermore, we wanted to delve more deeply into the relationship between closeness, empathy, and moral justification. Specifically, we wanted to test if the propensity to use justification strategies when the perpetrator is close could be explained by feeling less empathy toward the victim (H3a) and feeling greater empathy toward the perpetrator (H3b). To do so, we conducted a mediation analysis using the PROCESS macro by Hayes (2022) for SPSS (with 10,000 bootstrapping samples). We employed Model 4, incorporating empathy toward the victim and empathy toward the perpetrator as parallel mediators.

**Figure 1:**
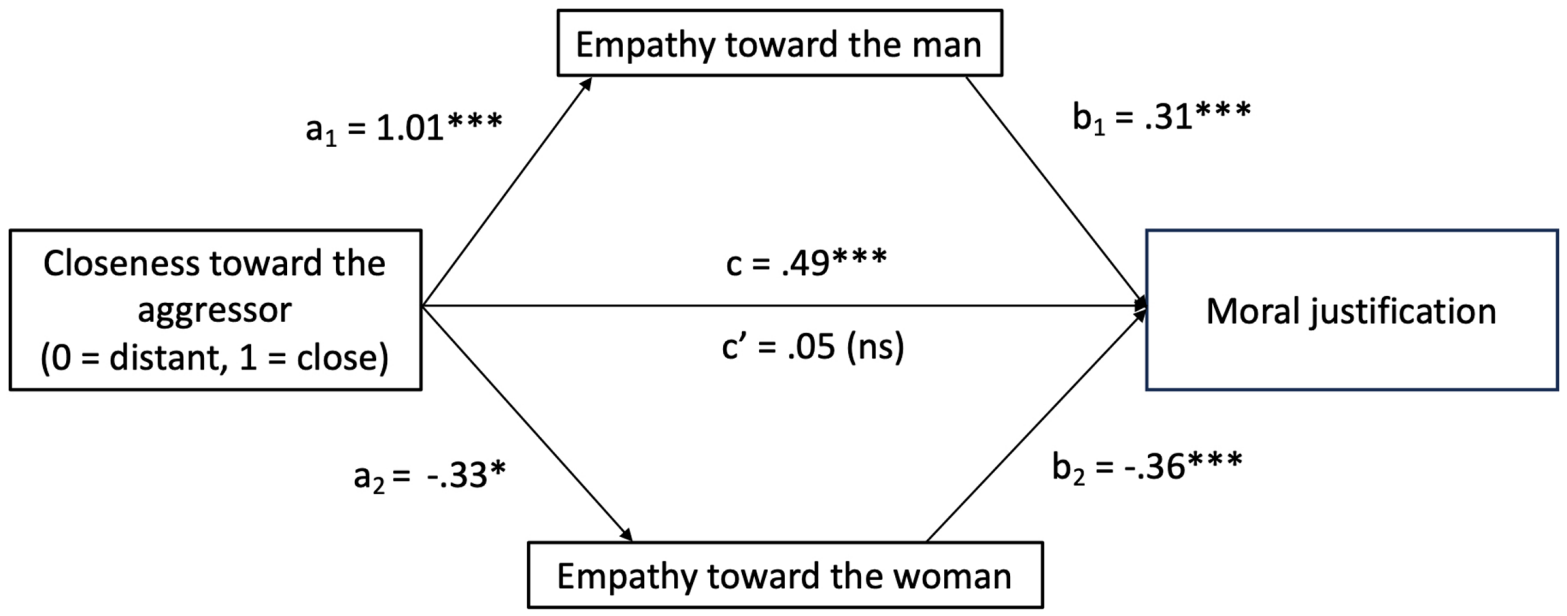
The mediation model of empathy toward the man and the woman in the relationship between closeness toward the aggressor and moral justification. *p < 0.026; ***p < 0.001; ns, non-significant.

As Figure 1 shows, being close to the perpetrator is positively associated with feeling empathy toward him (a1 = 1.01, *p <* 0.001), which, in turn, is positively associated with using moral justification strategies (b1 = 0.31, *p <* 0.001). Moreover, being close to the perpetrator is negatively associated with feeling empathy for the victim (a2 = −0.33, *p =* 0.026), and feeling less empathy for the victim leads to the use of more moral justification strategies (b2 = −0.36, *p* < 0.001). When both mediators were entered into the model, the significant direct effect of closeness toward the perpetrator on moral justification became non-significant (*c*’ = 0.05, *p =* 0.62), suggesting full mediation. Moreover, the total effect of closeness toward the perpetrator on moral justification was significant (*c* = 0.49, *p <* 0.001).

The total indirect effect of closeness to the perpetrator on moral justification was significant (*B =* 0.44, SE = 0.08, 95% CI [0.27, 0.62]). Specifically, closeness to the perpetrator indirectly affected moral justification through the mediating pathway of empathy toward the perpetrator (*B* = 0.31, SE = 0.06, 95% CI [0.20, 0.46]) and empathy toward the victim (*B* = 0.12, SE = 0.05, 95% CI [0.01, 0.02]). Therefore, we found support for H3. The use of moral justification when the perpetrator is close to the participants is explained by feeling more empathy toward the perpetrator and feeling less empathy toward the victim.

#### 3.3.2 Perpetrator dehumanization

We conducted a factor analysis to determine whether items were distributed across the theoretical dimensions of agency and experience (Gray et al., 2007). We found that memory was redistributed from agency to experience. Therefore, we reorganized the items, with agency now comprising self-control and morality (*α_agency_* = 0.87) and experience including hunger, fear, pain, and memory (*α_experience_* = 0.77). These results are consistent with other studies that also observed this equal item distribution (Borges-Castells et al., 2024).

To test the results of perpetrator dehumanization, we carried out an ANOVA 2 (closeness to the perpetrator: close vs. distant) × 3 (empathy focus: perpetrator vs. victim vs. control) for the agency dimension. We found a significant main effect of closeness to the perpetrator (*p* < 0.001). Participants dehumanized the perpetrator less when he was close rather than distant. We also found a significant main effect of empathy focus (*p =* 0.028). When empathy was focused on the perpetrator, participants significantly dehumanized him less than when the empathy was focused on the victim. There were no significant differences between the control condition and when the empathy was focused on the perpetrator (*p =* 0.1) or the victim (*p* = 0.098). The ANOVA statistics, as well as the means and standard deviations of the main effects, can be found in Table 1. We did not find significant interactions between closeness to the perpetrator and empathy focus.

These results support H1a, which expected that when the perpetrator was close with the participants, regardless of the empathy focus, participants would dehumanize him less. However, the results do not align with H2b, which expected that when the perpetrator was distant and the empathy was focused on him, he would be less dehumanized. This is because the interaction between empathy and closeness was not significant.

Given the similarity of these results to those related to moral justification, we decided to investigate the relationship between closeness to the perpetrator, empathy, and dehumanization through a non-pre-registered mediation. This analysis would enable us to examine whether empathy, like moral justification, served as a mediator in the relationship between closeness to the perpetrator and his dehumanization. We carried out a mediation analysis using the PROCESS macro by Hayes (2022) for SPSS (with 10,000 bootstrapping samples). We employed Model 4, incorporating empathy toward the victim and empathy toward the perpetrator as parallel mediators.

**Figure 2:**
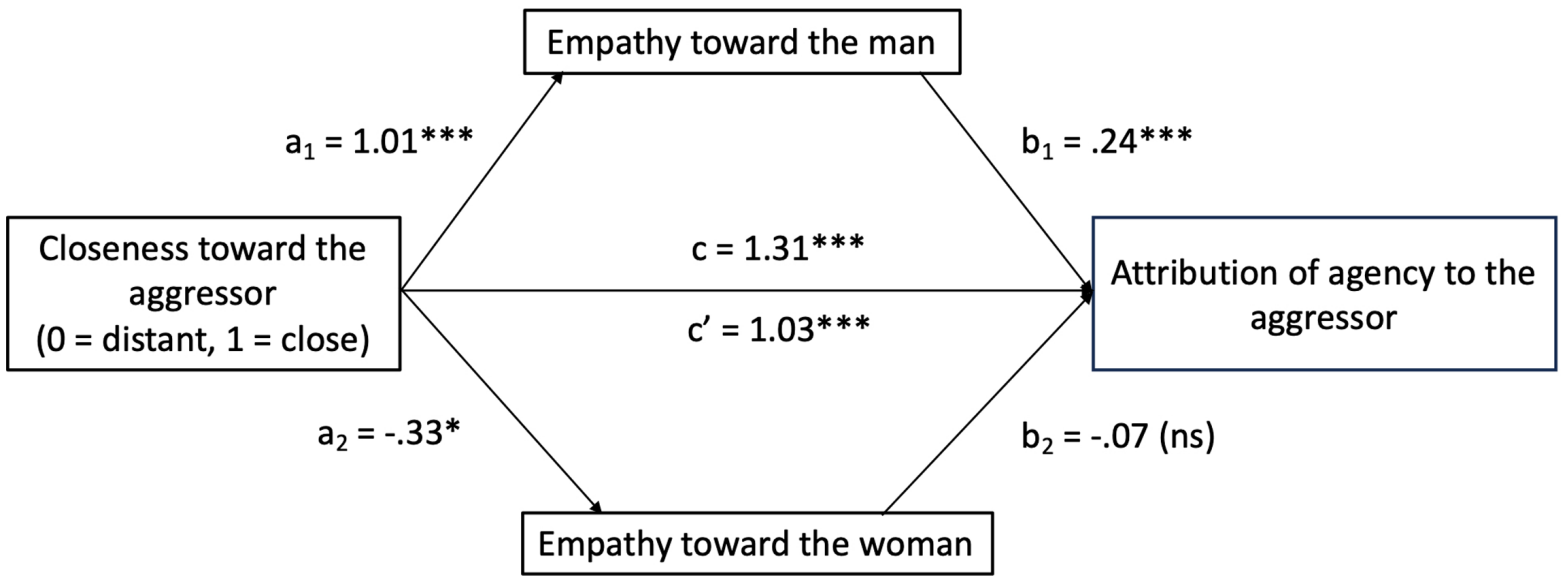
The mediation model of empathy toward the man in the relationship between closeness toward the aggressor and aggressor dehumanization. *p = 026; ***p < 0.001; ns, non-significant.

As Figure 2 shows, being close to the perpetrator is positively associated with feeling empathy toward him (a1 = 1.01, *p <* 0.001), which, in turn, is positively associated with the attribution of agency (b1 = 0.24, *p <* 0.001). Meanwhile, being close to the perpetrator is negatively associated with feeling empathy for the victim (a2 = −0.33, *p =* 0.026), and feeling empathy for the victim is negatively associated to attributing agency (b2 = −0.07, *p =* 0.24). Furthermore, the direct effect of closeness toward the perpetrator on agency attribution was significant (*c*’ = 1.03, *p <* 0.001), as well as the total effect (*c* = 1.31, *p <* 0.001).

Moreover, the total indirect effect of closeness to the perpetrator on the attribution of agency was significant (*B* = 0.27, SE = 0.08, 95% CI [0.11, 0.45]). Specifically, closeness to the perpetrator indirectly affected the attribution of agency, primarily mediated by empathy toward the man (*B =* 0.24, SE = 0.08, 95% CI [0.09, 0.43]), and it was not found to be statistically significant through empathy toward the woman (*B =* 0.02, SE = 0.02, 95% CI [−0.01, 0.08]). These results suggest that attributing agency to the perpetrator when the perpetrator is close to the participant is explained by feeling greater empathy toward him. When the perpetrator is close, there is a greater sense of empathy toward him; therefore, more agency is attributed to him, meaning that he is less dehumanized.

#### 3.3.3 Victim dehumanization

We conducted a factorial analysis to ascertain whether the items were distributed consistently with the dehumanization of the perpetrator. However, we initially obtained a single factor. Subsequently, we repeated the factorial analysis with the criterion of extracting two factors, resulting in a congruent distribution of items. Self-control and morality constituted one factor (agency; *α_agency_* = 0.80), while hunger, fear, pain, and memory comprised a second factor (experience; *α_experience_* = 0.80).

To test the results of victim dehumanization, we performed an ANOVA 2 (closeness to the perpetrator: close vs. distant) × 3 (empathy focus: perpetrator vs. victim vs. control) for the agency dimension. We did not find a significant main effect of closeness to the perpetrator (*p =* 0.77). There were no differences in dehumanizing the victim depending on closeness to the perpetrator. We did find significant differences on the empathy focus (*p =* 0.008). When empathy is focused on the perpetrator, participants dehumanize the victim more than when empathy is focused toward the victim (*p =* 0.009) or in the control condition (*p* = 0.049). There were no differences in dehumanizing the victim between the condition of empathy focus on the victim and the control condition (*p =* 0.1). The ANOVA statistics, as well as the means and standard deviations of the main effects, can be found in Table 1. The interaction between closeness to the perpetrator and the empathy focus was not significant (*p* = 0.666).

These results do not support any of our hypotheses. H1c anticipated that participants would dehumanize the woman more when the perpetrator was close, regardless of the focus of empathy. However, no differences were found based on closeness. Moreover, H2c expected that when the perpetrator was distant, the focus of empathy would have an effect; specifically, when empathy was focused on the perpetrator, the victim would be more dehumanized. However, we did not find the necessary interaction to confirm this hypothesis. We only observed that empathy is what determines greater or lesser dehumanization toward the victim, regardless of closeness.

### 3.4 Gender and sexism

Finally, we conducted exploratory analyses to understand the influence of specific individual factors such as gender and sexism on moral justification and dehumanization. Concerning sexism, our aim was to test whether the relationship between closeness and empathy with the dependent variables was affected by participants’ level of sexism. To address this question, we conducted partial correlations and compared the correlations with and without controlling for the effect of sexism. We observed that the correlations between closeness and empathy with dependent variables remained statistically significant even after controlling for the effect of sexism, suggesting that these relationships persist regardless of the level of sexism present in the sample. Additionally, the magnitudes of the correlations barely varied, indicating that the introduction of sexism did not significantly alter these relationships. These correlations are available in the Supplementary material.

Regarding gender, we performed several ANOVAs to investigate potential differences in participants’ responses based on their gender. We only found a significant main effect of gender in moral justification: *F*(344,1) = 7.24, *p =* 0.007, *η*_p_^2^ = 0.021. Specifically, men (*M* = 2.44, *SD* = 0.086) showed a higher use of moral justification strategies compared to women (*M* = 2.10, *SD* = 0.091).

## 4 Discussion

The research aimed to examine how closeness to the perpetrator and empathy—toward the perpetrator or toward the victim—influenced moral justification and the dehumanization of both the perpetrator and the victim. First, we expected that when the perpetrator was close to the participant, regardless of the empathy focus, participants would justify the perpetrator’s behavior more (H1a), dehumanize the perpetrator less (H1b), and dehumanize the victim more (H1c). Our findings provided support for H1a and H1b. Notably, when participants maintained a close relationship with the perpetrator, they were more inclined to justify the perpetrator’s behavior and dehumanize the perpetrator less, irrespective of the empathy focus. Both results align with what has been found in other studies (Borges-Castells et al., 2024). This suggests that closeness to the perpetrator is a critical factor influencing leniency toward harassment. However, regarding victim dehumanization, we did not find that significant effect for closeness to the perpetrator, therefore rejecting H1c. Participants dehumanized the victim to the same extent regardless of their closeness to the perpetrator. We observed that the only factor affecting the victim’s dehumanization was the empathy focus: when the empathy focus was on the victim, participants dehumanized her less. This result is crucial, as it shows an avenue for avoiding and reducing the harmful process of dehumanizing the victim. Also, it aligns with other research demonstrating the positive effects of fostering empathy toward victims (Schewe and O’Donohue, 1993; Decety and Cowell, 2014; Gevers and Dartnall, 2014). Furthermore, it is important to note that, while the hypothesis related to moral justification is supported (H1a), the values obtained are below the midpoint of the scale. This suggests that participants do not widely embrace moral justification strategies. However, despite these values being relatively low, group differences have been observed, thus supporting the hypothesis that moral evaluations of harassment behaviors vary depending on who the perpetrator is and where empathy is directed.

Second, we hypothesized that when the perpetrator had a distant relationship (vs. close), differences would emerge depending on the empathy focus. Specifically, we expected that when empathy was directed toward the perpetrator (vs. the victim), participants would justify the perpetrator’s behavior more (H2a), dehumanize the perpetrator less (H2b), and dehumanize the victim more (H2c). However, none of the hypotheses were supported. In all cases, it was evident that closeness to the perpetrator and the focus of empathy were two determinant factors for the dependent variables, considered individually rather than in their interaction.

Third, we expected that the propensity to use justification strategies when the perpetrator was close could be explained by feeling less empathy toward the victim (H3a) and feeling greater empathy toward the perpetrator (H3b). We found support for this hypothesis through a total mediation. Empathy acted as a mediator in the relationship between the perpetrator’s closeness to the participant and moral justification. When the perpetrator was close to the participant, there was increased empathy toward the perpetrator and decreased empathy toward the victim, a dynamic that enhanced the use of moral justification strategies. This result highlights the complexity of the relationship between closeness and empathy.

Upon discovering this outcome regarding moral justification, we opted to conduct a non-pre-registered analysis focusing on the dehumanization of the perpetrator. Through this analysis, we confirmed that empathy with the perpetrator served, again, as a mediator in the relationship between closeness to the perpetrator and dehumanization of the perpetrator. Therefore, we conclude that empathy serves as a key component shaping the cognitive interpretation of harassment in the context of a close relationship with the perpetrators. These results indicate that people being more lenient toward harassment perpetrated by close individuals is not a direct relationship; rather, it is mostly mediated by the empathy felt toward those perpetrators. In the case of moral justification, the increased moral justification when the perpetrators are close is mediated by experiencing greater empathy toward the perpetrator and reduced empathy toward the victim. Moreover, the decreased dehumanization of the perpetrator when he is close is mediated by experiencing greater empathy toward the perpetrator. Therefore, we can conclude that the defense of close perpetrators is a more intricate process than what has been previously elucidated. These results also highlight the relevance of considering empathy toward perpetrators.

In summary, this research demonstrates that both closeness to the perpetrators and the empathy focus are two highly relevant variables for the fight against harassment. Regarding closeness, participants being close to the perpetrator leads to the use of more moral justification strategies and less dehumanization of the perpetrator. These results are problematic because they suggest that harassment is less morally problematic when the perpetrator is close. The way the community sees gender-based violence can significantly shape how people respond to it—whether it is committed, whether the victim reports it, and whether a third party denounces it (Waltermaurer, 2012). Therefore, if the members of a community justify and defend close perpetrators, it is more likely that these aggressions continue to take place. Regarding empathy focus, favoring empathy toward the perpetrator vs. toward the victim results in different responses. On the one hand, when empathy is focused on the victim, there is less moral justification, the perpetrator is dehumanized more, and the victim is dehumanized less. On the other hand, when empathy is focused on the perpetrator, there is greater moral justification, the perpetrator is dehumanized less, and the victim is dehumanized more. This clearly reflects that empathy functions as a double-edged sword, as it can have both positive and negative effects.

Finally, participants’ sexism and gender are not variables that significantly affect the results. Sexism did not yield significant differences in the relationship between closeness and empathy with moral justification and the dehumanization of the perpetrator and the victim. The only significant gender difference was found in moral justification, with men employing more moral justification strategies than women. This aligns with existing research indicating gender disparities in justifying gender violence. For instance, men commonly use strategies such as offering the benefit of the doubt, interpreting situations with bias, or dismissing allegations as untrue (McDonald et al., 2010). Additionally, they often emphasize contextual factors to diminish the perpetrator’s responsibility, such as considering instances of rape as being more consensual when alcohol is involved (Novo et al., 2015). Men also tend to assign more blame to victims of sexual harassment compared to women (Bongiorno et al., 2020).

### 4.1 Practical implications and future directions

Given the findings of this research, it is essential to consider three fundamental aspects. First, it is crucial to recognize the importance of considering closeness as a key variable in the fight against gender violence. Closeness to perpetrators can foster a more benevolent view of harassment and its justification, ultimately posing a barrier to its eradication. Acknowledging and addressing this factor could allow us to move toward more effective interventions. Second, the promotion of empathy toward victims is important. This idea is already widely accepted in the scientific literature, which typically focuses on the role of the victim and the associated benefits of fostering empathy toward them in the fight against gender-based violence (Schewe and O’Donohue, 1993; Gevers and Dartnall, 2014).

Third, it is crucial to acknowledge a significant but less common finding: the negative consequences of empathizing with perpetrators. This concept is gaining importance in the scientific community, with research indicating that studying empathy toward perpetrators is as crucial as examining empathy toward victims (Diehl et al., 2014; Bongiorno et al., 2020). Interventions addressing gender-based violence should incorporate this perspective and recognize that empathizing with the perpetrator may lead to greater tolerance of harassment. Therefore, these interventions should channel empathy toward aggressors in a manner that does not promote tolerance of harassing behavior. Moreover, this could also be a factor to consider in the media. Media, including social and mass media, play a pivotal role in shaping societal beliefs and behaviors, making it crucial to carefully control how gender-based violence is portrayed. For instance, evidence indicates that media outlets engaging in the sexual objectification of women contribute to the normalization of harassment behaviors, thus impacting instances of sexual harassment through various cognitive and emotional mechanisms (Galdi and Guizzo, 2021). Furthermore, a meta-analysis has revealed a significant relationship between exposure to pornography and the acceptance of rape myths (Hedrick, 2021). Given these insights, it is imperative to critically assess how gender-based violence and its perpetrators are presented in the media. Specifically, considering that this study has revealed the negative consequences of empathizing with the perpetrators, it is essential to monitor how they are portrayed in the media. At times, they can be excessively humanized by telling their stories, focusing on personal aspects, or presenting their remorse in isolation. If not handled with care, this approach could—as seen in this research—lead to greater leniency. Therefore, the importance lies in maintaining an informative balance that considers both the perspectives of perpetrators and victims, thus avoiding distortions that could undermine a comprehensive understanding of the facts and their consequences.

In summary, while existing literature has acknowledged the significance of empathy, it has not extensively investigated factors contributing to its development, particularly the dynamics within the perpetrator’s social circle. Our study’s findings reinforce prior research indicating that empathy toward the perpetrator poses a challenge in addressing gender-based violence (Diehl et al., 2014; Bongiorno et al., 2020). Furthermore, we introduce a crucial factor: the closeness of individuals to the aggressors. Our research highlights that feelings of empathy toward the perpetrator intensify when observers of violence are closely associated with the perpetrator.

In terms of future research, it would be crucial to develop effective strategies to reduce empathy toward perpetrators of gender-based violence who are closely connected to the individual. This may involve exploring psychosocial intervention techniques aimed at addressing the positive perception of aggressors within close circles.

Furthermore, this study has demonstrated that adopting an empathetic and humanized perspective toward the aggressor can have negative consequences on both the justification and dehumanization of both the perpetrator and the victim. It would be important to investigate how these portrayals influence other aspects of societal attitudes and behaviors toward the victim and the aggressor. For instance, it would be relevant to examine how support or confrontation from the environment toward the aggressor affects intentions to help the victim.

Moreover, in line with the notion that empathizing with and humanizing the perpetrator led to a more lenient response toward harassment, it would be pertinent to delve deeper into this humanization of the aggressor and directly explore what happens when the perpetrator is humanized. Research often focuses on the dehumanization of victims (Cuddy et al., 2007; Bastian and Haslam, 2011) and aggressors (Bastian et al., 2013; Khamitov et al., 2016). Therefore, it would be relevant for future research to explore the effects of humanizing aggressors, including how it influences attribution of responsibility and perception of violent behavior, as well as how these perceptions may impact societal responses.

### 4.2 Limitations

The findings of the current study should be interpreted cautiously, as there are certain limitations inherent in this research. First, we decided to focus more specifically on the perpetrator, considering only the participants’ closeness to the perpetrator and not to the victim. This emphasis on the perpetrator’s closeness, without considering the victim’s, may limit a comprehensive understanding of harassment dynamics. Exploring closeness to the victim in future studies could further enrich the research, providing a more complete insight into how relationships impact responses to harassment. Second, due to the absence of a moral disengagement scale suitable for use in the specific context of manipulation, we had to develop our measure based on a theoretical model (Bandura, 1991, 2011)—a practice adopted by other authors facing the same challenge (Caprara et al., 2009; Page et al., 2016). While this method aligns with common procedures, the lack of a psychometrically validated scale may introduce methodological concerns. Third, we only utilized a single behavior related to online gender-based violence. It would be relevant to examine whether the same results can be replicated with a larger number of behaviors and with behaviors from other categories, such as offline gender-based violence, verbal, physical, and sexual aggression. Exploring a wider range of behaviors in future studies could offer a more nuanced understanding of the issue.

## 5 Conclusion

Movements like “Sister, I do believe you” aim to emphasize the importance of supporting victims of gender-based violence. However, societal responses to gender-based violence often lean toward blaming the victim more than the perpetrator. This research has shown that two initially perceived positive factors—closeness and empathy—can blur moral lines, facilitating a social response that supports perpetrators rather than defending victims. Specifically, this study reaffirms that individuals tend to show greater leniency toward perpetrators of gender harassment when they are part of their close circle. Empathy emerges as a key factor that directly influences the inclination to be more or less lenient depending on who it is directed toward. Empathy toward the victim acts as a protective factor, reducing leniency, while empathy toward the perpetrator intensifies it. Furthermore, the use of moral justification strategies for close perpetrators is mediated by higher empathy for the perpetrator and lower empathy for the victim. Similarly, reduced dehumanization of close perpetrators is mediated by increased empathy for the perpetrator. In both cases, heightened empathy toward the perpetrator is crucial, as it fosters greater tolerance toward harassment. Further research in this direction could lead to the discovery of new approaches for interventions against harassment and gender-based violence, ultimately contributing to their eradication.

## Data availability statement

The datasets presented in this study can be found in online repositories. The names of the repository/repositories and accession number(s) can be found in the article/Supplementary material.

## Ethics statement

The studies involving humans were approved by Committee on Research and Animal Welfare of the University of La Laguna (CEIBA2023-3257). The studies were conducted in accordance with the local legislation and institutional requirements. The participants provided their written informed consent to participate in this study.

## Author contributions

NB-C: Conceptualization, Data curation, Formal analysis, Investigation, Methodology, Project administration, Resources, Validation, Visualization, Writing – original draft, Writing – review & editing, Software. VB: Conceptualization, Funding acquisition, Investigation, Project administration, Resources, Supervision, Validation, Visualization, Writing – review & editing. AR-P: Conceptualization, Data curation, Funding acquisition, Investigation, Project administration, Resources, Supervision, Validation, Visualization, Writing – review & editing.
