# Supplementary material for: Understanding the support for gender-based harassment perpetrators: the role of closeness and empathy
Source: Front Psychol. 2024 Jun 27;15:1418404. doi: 10.3389/fpsyg.2024.1418404 (PMC11236737; doi:10.3389/fpsyg.2024.1418404)
Supplement: Supplementary file 1 [file Table_1.docx]

Supplementary Material

# Supplementary Data

Supplementary material, along with the databases, can be found at: <https://osf.io/whta4/>
